# Prognostic Role of Visual Evoked Potentials in Non-Neuritic Eyes at Multiple Sclerosis Diagnosis

**DOI:** 10.3390/jcm12062382

**Published:** 2023-03-20

**Authors:** Domizia Vecchio, Paolo Barbero, Giulia Galli, Eleonora Virgilio, Paola Naldi, Cristoforo Comi, Roberto Cantello

**Affiliations:** 1 Neurology Unit, Department of Translational Medicine, Maggiore della Carità Hospital, University of Piemonte Orientale, Corso Mazzini 18, 28100 Novara, Italy; 2 Interdisciplinary Research Center of Autoimmune Diseases (IRCAD), Department of Health Sciences, University of Piemonte Orientale, Corso Mazzini 18, 28100 Novara, Italy; 3 Phd Program in Medical Sciences and Biotechnologies, Department of Translational Medicine, University of Piemonte Orientale, Corso Mazzini 18, 28100 Novara, Italy

**Keywords:** visual evoked potentials, multiple sclerosis, non-neuritic eye, P100 latency, prognosis, disability outcome

## Abstract

***Introduction***: This study aimed to assess the prognostic role of visual evoked potentials (VEPs) of the non-neuritic eye at the diagnosis of multiple sclerosis (MS). ***Patients and methods***: We enrolled 181 MS patients (62% females, mean age at diagnosis: 38 years, standard deviation: 12) at the time of the first diagnostic work-up, including VEPs. We collected P100 latency and N75-P100 amplitude of non-neuritic eyes at diagnosis, and then we calculated the mean values in 127 patients with no history of optic neuritis (ON) or considered the unaffected eye in the remaining. At last follow-up (minimum: one year), disability was evaluated according to MS Severity Score or MSSS (median: 2.44, range: 0.18–9.63). Statistical analysis included Mann–Whitney descriptive analysis, Spearman correlation for independent samples, and linear regression for significant predictors of MSSS. ***Results***: 38/181 patients had P100 latency >115 ms, and 63/181 showed N75-P100 amplitude < 5 microV in the unaffected eyes at MS diagnosis. At last follow-up, MSSS correlated with P100 latency (rho = 0.21, *p* = 0.004) and N75-P100 amplitude (rho = 0.19, *p* = 0.009) collected at diagnosis. P100 latency (not N75-P100 amplitude) resulted in a predictor for disability over time (MSSS) in the regression model (along with age at onset, MS course, and disease-modifying treatments). ***Conclusions***: Our study showed a prognostic value of VEPs in clinically unaffected eyes at MS diagnosis to predict future disability, independently from a history of ON.

## 1. Introduction

Multiple sclerosis (MS) is an inflammatory autoimmune disease of the nervous system in which neurodegeneration occurs at early stages and relates with the accumulation of disability. The visual system emerged as a model for detecting both inflammation and axonal loss in the subclinical phase [1]. Despite the fact that optic neuritis (ON) affects up to 70% of MS patients during the disease course, visual evoked potentials (VEPs) could detect early damage also in the clinically unaffected eye (non–ON) [2]. In fact, the latency of the P100 waveform peak, VEP main component, reflects mostly myelin integrity through demyelination and remyelination, and the amplitude of the N75-P100 waveform represents axonal dysfunctions caused by transient conduction blocks or optic nerve permanent damage [3].

Our study aims to evaluate the prognostic role of subclinical abnormalities of the visual pathway at disease onset. We thus related VEP components, derived from non-ON eyes at the diagnosis, to disability over time. 

## 2. Patients and Methods

We collected data from 181 MS patients (62% females, mean age at diagnosis: 35.4 years ± standard deviation or SD 11.3), who performed VEPs at their first diagnostic work-up. At that time, mean disease duration from clinical onset was 1.4 years (±standard error or ES 0.3). Patients were subsequently recruited from January 2016 to December 2021 at the University of Piemonte Orientale “Maggiore della Carità” Hospital in Novara and at the “San Andrea” Hospital in Vercelli. Patients with other inflammatory and neurodegenerative diseases, psychiatric or ophthalmological comorbidities were excluded. 

Minimum follow-up from diagnosis was one year (mean 2.6 years ± ES 0.2). At last clinical evaluation, we evaluated their outcome, according to MS Severity Score (MSSS), and collected disease-modifying treatments (DMTs), grouped as follows: none, low–moderate (injective therapies, dimethyl fumarate, teriflunomide), or highly effective (sphingosine-1-phosphate receptor inhibitors, natalizumab, depletive therapies). 

Pattern reversal VEPs were recorded according to the International Society for Clinical Electrophysiology [4]. Full-field monocular stimulation was obtained using a high-contrast black-and-white checkerboard spanning the central 30° of the visual field and reversed at a rate of 1.5–2/s (Galileo Systems-Be Light, EBNeuro). The P100 peak latency and the N75-P100 peak-to-peak amplitude were bilaterally examined for all subjects. Normal cut-offs were defined as: P100 latency < 115 ms, and N75-P100 amplitude > 5 microV [4]. We mainly analyzed clinical unaffected eyes. Thus, we calculated the mean values of VEP components for both sides in 127 patients with no history of ON, and included data measured only in the unaffected eye in the remaining 54 cases with ON. 

Statistical Analysis was performed using SPSS 25.0 for Windows (SPSS Inc., Chicago, IL, USA). We verified whether the data were distributed according to the Gaussian model with the Kolmogorov–Smirnov test and consequently used non-parametric tests: Mann–Whitney U for comparison between continuous variables; Fisher test for categorical variables. The Spearman’s rank correlation coefficient test was used for the correlation between continuous variables. Linear regression analysis was performed for disability according to MSSS (dependent variables are discussed in the Results section). All tests were two-sided and the significance threshold was set to *p* < 0.05.

## 3. Results

We collected 166 relapsing-remitting (RR) MS, and 15 progressive patients (5 primary progressive and 10 secondary progressive MS; these latter were not investigated at first attacks). Clinical–demographic data at the diagnostic work-up are shown in Table 1. ON was the onset attack in 54 (30%) cases, of whom 11 (20%) patients showed no VEP responses in the affected eye, and 43 (80%) a prolonged P100 latency (mean 123.5 ms ± ES 3.0) with reduced wave amplitude (4.8 µV ± 0.5). 

Considering VEP responses collected at diagnosis in non-ON eyes, 38/181 patients presented a P100 latency > 115 ms, and 63/181 a N75-P100 amplitude < 5microV (mostly if responses were delayed; *p* < 0.001). Of note, a history of ON did not relate with abnormal VEP responses in the fellow eye. Males (*p* = 0.004) and patients with progressive MS (*p* = 0.01) showed more frequently pathological VEPs recorded from non-neuritic eyes.

**Figure 1:**
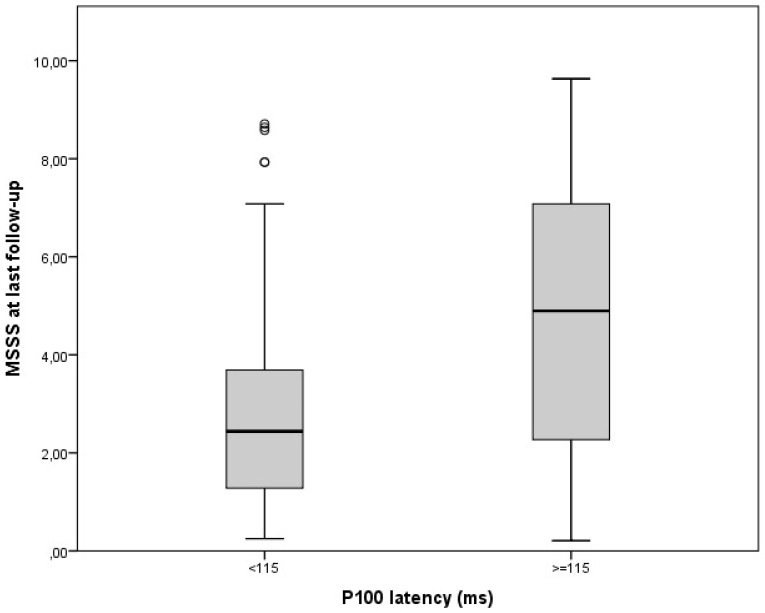
Disability at last follow-up according to baseline P100 latency in non-neuritic eyes (*p* = 0.004).

**Figure 2:**
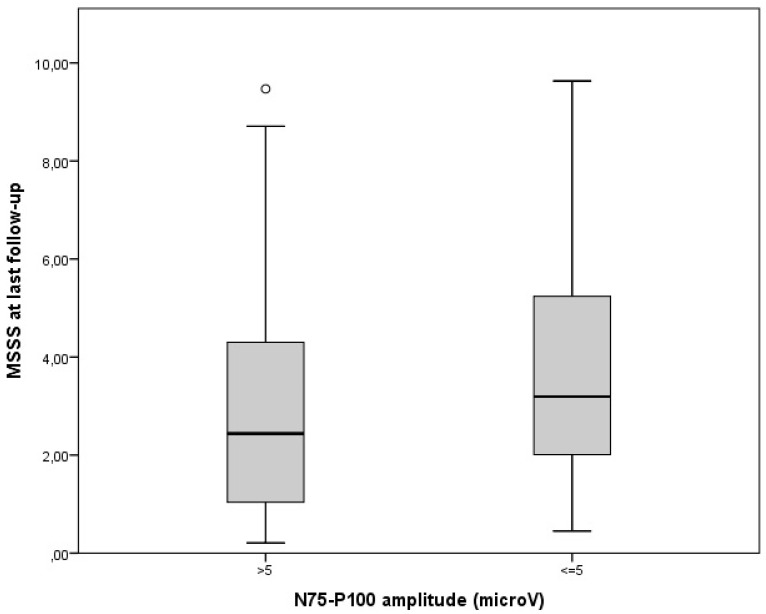
Disability at last follow-up according to baseline N75-P100 amplitude in non-neuritic eyes (*p* = 0.009).

Disability measures were collected at last follow-up and varied largely among our cohort (median EDSS was 1.5, range 1–8; MSSS 2.44, range: 0.18–9.63). MSSS correlated with age at onset (rho = 0.30 *p* < 0.001) and progressive MS course at diagnosis (rho = 0.31 *p* < 0.001), both established MS prognostic factors [5]. Nonetheless, patients with abnormal VEPs (P100 latency > 115 ms or N75-P100 amplitude < 5microV) in the non-ON eyes at diagnosis showed a higher disability at last follow-up (Figure 1 and Figure 2). In fact, both wave latency (rho = 0.21, *p* = 0.004) and amplitude (rho = 0.19, *p* = 0.009), that were recorded at the unaffected eye at diagnostic work-up, correlated with MSSS. 

Subsequently, we performed a multivariate analysis that included last MSSS as the dependent variable; gender, age at onset, disease course (RRMS/progressive) at diagnosis, the history of previous ON (yes/no), and VEP measures at baseline as dependent variables. P100 latency (not N75-P100 amplitude), collected at diagnosis in the non-ON eye, emerged as a mild predictor for disability over time (MSSS) (odd risk or OR: 0.04; 95% confidence interval or CI: 0.01–0.06; *p* = 0.002). Age at onset and disease course were also confirmed as disability predictors (Table 2A). 

Secondly, we limited the analysis to RRMS patients (N = 166), and confirmed previous results for P100 latency at last follow-up (OR for P100 latency in the non-ON eye: 0.04; 95% CI: 0.01–0.6; *p* = 0.005) (Table 2B). In this cohort, we also evaluated if DMT initiation related to disability. In fact, 33/166 RRMS patients remained treatment-naïve at the end of their follow-up, whereas 132/166 commended a DMT (low–moderate effective in 107 patients and highly effective in 25). Including DMTs in our multivariate analysis, not only treatments resulted as significant predictors for disability, but also the prognostic role for age at onset and VEP latency was confirmed (Table 2B). Of note, treatment-naïve patients were mainly those with a first clinical event and oligoclonal bands in the cerebrospinal fluid, who remained stable to radiological and clinical monitoring, and few who refused therapy.

## 4. Discussion

Our study suggested a prognostic role for VEPs at MS diagnosis independently from the clinical history of visual involvement. In fact, abnormal P100 latency in the unaffected eyes emerged as a marker for future disability. Moreover, age at onset, MS course, and DMTs were confirmed as predictors for MSSS in our cohort. 

To prevent damage accumulation, an early personalized DMT should be discussed since MS onset and according to risk factor evaluations. Patients with more severe RRMS need to start highly effective therapies at diagnosis. To identify those cases, conventional magnetic resonance imaging (MRI) is considered the most effective tool to evaluate disease burden, whereas it is not that sensitive for visual pathways [6]. On the contrary, VEPs provide easily detectable and functional measures of the optic system [2]. Few studies, mostly including multimodal registrations, related prolonged responses to disability. In fact, visual and motor evoked latencies at baseline were correlated to disability, according to EDSS, at 2 years in 30 patients [7]. Moreover, if considering multimodal evoked potentials (EPs), abnormal responses at diagnosis were related to prognosis at several timepoints in different studies: to EDSS > 3.5 at 5 years in 50 patients [8], to disability progression in 6 years in 100 patients [9], to a higher EDSS at 20 years in 28 patients independently from baseline lesion load and disability [10]. Finally, London at al. evidenced a higher risk of reaching a EDSS milestone of 4 or 6 at 10 years according to multimodal EP involvement at diagnosis in 108 cases [11]. 

We decided to focus on the optic pathway, since it was not affected by spinal damage. Regarding the visual system, Leocani et al. [12] reviewed the prognostic role of VEPs in small cohorts. VEP latency correlated with disability progression at 8 years in 37 patients treated with interferon beta-1a. This cohort was tested cross-sectionally at the end of the follow-up, and 42% showed prolonged P100 latencies despite no history of ON. Longitudinal data were available in 16 patients [13]. In our study, we enrolled a large sample of newly diagnosed MS cases before DMT initiation. 

Another strength of our study was to focus on subclinical visual damage. In fact, 21% of our 181 patients at diagnosis showed a prolonged P100 latency in non–ON eyes, and irrespectively from a history of contralateral ON. Seven (18%) patients with abnormal VEPs in clinically unaffected eyes showed a progressive MS course at diagnosis, according to previous data [14]. A role of VEPs in progressive MS was suggested by Sater et al. that presented P100 latency as a tool for monitoring disease course in 11 patients [15]. Regarding repetitive measures over time, VEP parameters did not change significantly 2 years after baseline registration in 52 RRMS, including patients with a previous history of ON. In this latter cohort, baseline abnormal VEPs related to concomitant EDSS [16]. 

We decided to investigate the disability outcome in the short term using MSSS, to correct for disease duration. In our multivariate model, a delayed visual response in clinically unaffected eyes resulted as a mild predictor of disability. For this reason, we suggested that P100 latency at baseline should be included in predictors of disability over time.

Our study has some limitations.

(1) We employed traditional full-field VEPs as an easily repeatable and largely used marker. Some authors evaluated multifocal (mf) VEPs in smaller groups and found some correlations with disability. Blanco et al. showed delayed and reduced visual responses in more than 70% of non-ON cases among a group of 28 patients. They also described lower wave amplitudes with higher disability scores [17].

Generally, mfVEPs are more specific and sensitive in detecting dysfunctions of specific parts of the visual field, but this technique is not standardized among centers and time-consuming to be performed [18].

(2) Since our study included a large cohort of 181 MS patients at diagnosis, our results could not reflect long-term treatment-related prognosis. The choice of starting highly effective DMTs could be considered as a marker of disease severity at diagnosis in our cohort.
(3) Pivotal data on optical coherence tomography we performed in a limited subgroup with no definite results. Nonetheless, VEPs are currently diffusely employed in MS neurological assessments at first MS diagnostic work-up.

Adding other biomarkers, such as neurofilaments, to our model could improve the prognostic value of VEP latency at diagnosis.

## 5. Conclusions

Our study showed a prognostic value of VEPs at MS diagnosis in predicting short-term disability, independently from a previous history of ON. 

P100 latency in non-ON eyes could be considered as a prognostic factor over time.

## Figures and Tables

**Table 1:** Clinical–demographic features of patients collected at the diagnostic work-up (N = 181).

Female (N, %)	113, 62%
Age at onset (years: mean, SD) Age at diagnosis (years: mean, SD)	35.4, 11.3 36.9, 11.7
MS course at diagnosis (N, %): relapsing-remitting progressive (primary or secondary)	166, 92% 15, 8%
Previous history of ON or at onset (N, %)	54, 30%
VEP measures in non-ON eyes at diagnosis: abnormal P100 latency (patients: N, %) P100 latency (ms: mean, ES) abnormal N75-P100 amplitude (patients: N, %) N75-P100 amplitude (microV: mean, ES)	38, 21% 106.4, 1.1 63, 35% 7.1, 0.3
Disease duration at diagnosis (years: mean, ES)	1.4, 0.3
EDSS at diagnosis (median, range)	1, 0–8

Legend: N: number; SD: standard deviation, MS: multiple sclerosis, ES: standard error, ON: optic neuritis, VEP: visual evoked potentials, EDSS: expanded disability status score.

**Table 2:** Multivariate analysis of clinical–demographic factors, evaluated at diagnosis, and disability over time, according to multiple sclerosis severity score.

Prognostic Factors	OR	IC 95%	*p*-Value	Overall *p*-Value
**A: N = 181 Patients (All Cases); R = 0.47, R-Square = 0.22, F-Value = 8**				
**Gender** **Age at onset** **Previous history of ON or at onset** **MS course** **P100 latency** **N75-P100 amplitude**	0.29 0.04 0.10 2.07 0.04 −0.04	−0.42–0.10 0.01–0.07 −0.62–0.82 0.80–3.33 0.01–0.06 −0.13–0.06	NS 0.005 NS 0.002 0.002 NS	<0.001
**B: N = 166 patients (RRMS); R = 0.35, R-square = 0.13, F-value = 4**				
**Gender** **Age at onset** **Previous history of ON or at onset** **P100 latency** **N75-P100 amplitude** **DMTs**	0.44 0.04 0.11 0.04 −0.05 0.11	−0.29–1.16 0.01–0.07 −0.61–0.82 0.01–0.06 −0.14–0.04 0.17–1.28	NS 0.025 NS 0.005 NS 0.010	0.001

**Legend:** N: number; OR: odd risk, IC: interval confidence, MS: multiple sclerosis, ON: optic neuritis, DMTs: disease-modifying treatments (DMTs), grouped as follows: none, low–moderate (injective therapies, dimethyl fumarate, teriflunomide) or highly effective (sphingosine-1-phosphate receptor inhibitors, natalizumab, depletive therapies).

## Data Availability

Data available on request.
